# Bacterial Diversity and Mycotoxin Reduction During Maize Fermentation (Steeping) for *Ogi* Production

**DOI:** 10.3389/fmicb.2015.01402

**Published:** 2015-12-15

**Authors:** Chiamaka A. Okeke, Chibundu N. Ezekiel, Cyril C. Nwangburuka, Michael Sulyok, Cajethan O. Ezeamagu, Rasheed A. Adeleke, Stanley K. Dike, Rudolf Krska

**Affiliations:** ^1^Department of Biosciences and Biotechnology, Babcock UniversityIlishan Remo, Nigeria; ^2^Microbiology and Environmental Biotechnology Research Group, Agricultural Research Council–Institute for Soil, Climate and WaterPretoria, South Africa; ^3^Department of Agriculture, Babcock UniversityIlishan Remo, Nigeria; ^4^Center for Analytical Chemistry, Department of Agrobiotechnology (IFA-Tulln), University of Natural Resources and Life Sciences ViennaTulln, Austria; ^5^Unit for Environmental Science and Management, North-West University at PotchefstroomPotchefstroom, South Africa; ^6^Department of Microbiology, Imo State UniversityOwerri, Nigeria

**Keywords:** diversity, ecology, fermentation, food safety, maize, mycotoxins, *ogi*

## Abstract

Bacterial diversity and community structure of two maize varieties (white and yellow) during fermentation/steeping for *ogi* production, and the influence of spontaneous fermentation on mycotoxin reduction in the gruel were studied. A total of 142 bacterial isolates obtained at 24–96 h intervals were preliminarily identified by conventional microbiological methods while 60 selected isolates were clustered into 39 OTUs consisting of 15 species, 10 genera, and 3 phyla by 16S rRNA sequence analysis. Lactic acid bacteria constituted about 63% of all isolated bacteria and the genus *Pediococcus* dominated (white maize = 84.8%; yellow maize = 74.4%). *Pediococcus acidilactici* and *Lactobacillus paraplantarum* were found at all steeping intervals of white and yellow maize, respectively, while *P. claussenii* was present only at the climax stage of steeping white maize. In both maize varieties, *P. pentosaceus* was found at 24–72 h. Mycotoxin concentrations (μg/kg) in the unsteeped grains were: white maize (aflatoxin B_1_ = 0.60; citrinin = 85.8; cyclopiazonic acid = 23.5; fumonisins (B_1_/B_2_/B_3_) = 68.4–483; zearalenone = 3.3) and yellow maize (aflatoxins (B_1_/B_2_/M_1_) = 22.7–513; citrinin = 16,800; cyclopiazonic acid = 247; fumonisins (B_1_/B_2_/B_3_) = 252–1,586; zearalenone = 205). Mycotoxins in both maize varieties were significantly (*p* < 0.05) reduced across steeping periods. This study reports for the first time: (a) the association of *L. paraplantarum*, *P. acidilactici*, and *P. claussenii* with *ogi* production from maize, (b) citrinin occurrence in Nigerian maize and *ogi*, and (c) aflatoxin M_1_, citrinin and cyclopiazonic acid degradation/loss due to fermentation in traditional cereal-based fermented food.

## Introduction

Maize (*Zea mays* L.) is a staple food in many parts of the world including sub-Saharan Africa (SSA). In Nigeria and some other West African countries, it is traditionally transformed by submerged fermentation to *ogi* – a complementary weaning food for infants and young children, convenient food for the sick, convalescent and elderly or quick breakfast mostly for those living in rural areas characterized by low income (Onyekwere et al., 1989; Steinkraus, 1996). *Ogi* is preferred by nearly 150 million West Africans (Oguntoyinbo and Narbad, 2012) mainly due to the ease of preparation of this gruel and the numerous associated nutritional benefits including high calorie, minerals, vitamins, and probiotic contents (Opere et al., 2012). The rich probiotic contents of *ogi* and other traditionally fermented cereal foods result from the indigenous beneficial microbial flora that play significant roles during cereal fermentation to yield the final product (Odunfa, 1985). Furthermore, fermentation provides a variety of foods and contributes to food preservation.

Fermentation during *ogi* production occurs in two distinct stages: (1) steeping of maize prior to obtaining *ogi* gruel and (2) souring of fermented *ogi* (Omemu, 2011). Several authors have reported on *ogi* production from various varieties of maize (white and yellow), from guinea corn, millet and sorghum after steeping for either 24, 48, 72, or 96 h (Odunfa and Adeyele, 1985; Teniola and Odunfa, 2002; Teniola et al., 2005; Adebayo and Aderiye, 2007; Adebayo-tayo and Onilude, 2008; Dike and Sanni, 2010; Omemu, 2011; Banwo et al., 2012; Oyedeji et al., 2013). Usual practice in the local/traditional setting, however, is that maize is steeped for at least 48 h and may extend to 96 h. In order to understand the ecology of species and promote biotechnology through beneficial strain selection for improved *ogi* production, microbial communities associated with spontaneous maize fermentation to *ogi* have been characterized and studied. The majority of the studies considered microbial diversity in mashed fermented maize grains (Teniola et al., 2005; Adebayo and Aderiye, 2007; Omemu, 2011) while others characterized fermenters from souring *ogi* samples (Odunfa and Adeyele, 1985; Teniola and Odunfa, 2002; Adebayo-tayo and Onilude, 2008; Oguntoyinbo et al., 2011; Banwo et al., 2012; Oguntoyinbo and Narbad, 2012); only Oyedeji et al. (2013) examined maize steep liquor of 24–72 h for fermenter diversity. From the aforementioned studies, *Lactobacillus* and *Leuconostoc* have been reported as the occurring genera of lactic acid bacteria (LAB) in maize steep liquor and fermented/souring *ogi* while *Pediococcus* was only reported in fermented/souring *ogi*.

Consumption of maize and maize-based food products is threatened by the presence of fungal toxins (e.g., aflatoxins, cyclopiazonic acid; fumonisins, and zearalenone; Warth et al., 2012; Abia et al., 2013a; Kayode et al., 2013; Adetunji et al., 2014a). Potential mycotoxin exposure through maize consumption has been identified to be higher in rural areas of developing countries where vulnerable consumers utilize broken and damaged kernels for diverse dietary purposes; such kernels are usually cheaper but could possibly be higher in mycotoxin content (Abia et al., 2013b; Njumbe Ediage et al., 2013; Adetunji et al., 2014b; Ezekiel et al., 2014). Efforts toward diversification of maize meals for enhanced nutritional benefits led to *ogi* production involving fermentation steps. There are suggestions that traditional fermentation can reduce mycotoxin transfer from grains to fermented foods whilst enhancing the nutrient content of food through the synthesis of protein, vitamins, and amino acids (Mokoena et al., 2005; Shetty and Jespersen, 2006; Juodeikiene et al., 2012).

Past studies with respect to *ogi* production focused mostly on studying microbial diversity and the roles of fermenters against natural microbial contaminants in *ogi* production (Odunfa and Adeyele, 1985; Teniola and Odunfa, 2002; Teniola et al., 2005; Adebayo and Aderiye, 2007; Adebayo-tayo and Onilude, 2008; Oguntoyinbo et al., 2011; Omemu, 2011; Banwo et al., 2012; Oguntoyinbo and Narbad, 2012; Oyedeji et al., 2013). There are also reports available on mycotoxin reduction mediated by single or combined cultures of LAB and other fermentation bacteria artificially inoculated into maize and other grains (Mokoena et al., 2005; Shetty and Jespersen, 2006; Oluwafemi and Da-Silva, 2009; Cho et al., 2010; Nyamete, 2013; Zhao et al., 2015). In spite of the aforementioned reports, there is no information on the influence of spontaneous fermentation mediated by autochthonous microbial communities interacting during *ogi* production on mycotoxin reduction in the gruel. Such information could provide important data to establish the relationship between fermentation and mycotoxin levels in *ogi*. This study therefore aims to discover the diversity and succession of bacteria during maize fermentation (steeping) into *ogi*, and to determine possible mycotoxin reduction due to fermentation influenced by indigenous microbial communities.

## Materials and Methods

### Source of Maize Grains and Preparation of *Ogi* Samples

Two maize varieties (white and yellow) were purchased from Ikenne market in Ogun State, Nigeria and used for this study. Grains of the white variety had been stored for less than one month while those of the yellow variety were approximately 6 months old in store. The grains were sorted to remove particulate matter and batched (750 g per batch) into the various groups based on the steeping durations (48, 72, and 96 h). Each batch was set up in triplicate, steeped in 1.5 L of clean tap water in large plastic bowls with lids and allowed to undergo spontaneous fermentation at ambient temperature. Steep liquor from the batches was taken at intervals of 24–96 h and bacteria associated with the steeping process were isolated and characterized. The pH of each steep liquor was determined at 24–96 h.

At the end of each steeping duration (48, 72, and 96 h), the remaining steep liquors were drained off and discarded. The fermented/softened maize grains were processed into *ogi* as previously described by Adebayo and Aderiye (2007). *Ogi* samples obtained from the various batches representing the steep durations were not left to sour but were immediately pressed to remove excess water and taken for mycotoxin analysis.

### Isolation of Bacteria from Maize Steep Liquor

Serial dilutions of steep liquor obtained from maize fermentation batches were performed and used for isolation of aerobic and anaerobic bacteria on plate count agar (PCA, Oxoid CM 325, Unipath, Hampshire, England) and deMann Rogosa Sharpe (MRS, Oxoid CM 361) agar, respectively (Haddadin et al., 2004). The pour-plate method was used for inoculation. MRS agar was supplemented with sodium azide (0.01%, Labtech Chemicals, Chinchwad Gaon, Pune, India) to inhibit aerobic bacteria and facilitate the growth of LAB (Cho et al., 2013). PCA plates were incubated for 24 h at 35°C for isolation of mesophilic aerobic bacteria while MRS agar plates were incubated for 24–72 h at 35–45°C in an Anaerobic Gas-Pack system Jung et al. (2012). In order to ensure good representation of isolates, all colonies were sampled from plates containing fewer than 10 colonies or five distinct colonies were randomly selected in colony dense plates. Isolates obtained were purified by a two-time repeated streaking on fresh agar plates of PCA and MRS, and maintained on same agar slants at 4°C at the Microbiology Laboratory of Babcock University, Nigeria.

### Identification of Bacterial Isolates

#### Morphological and Biochemical Identification

For preliminary identification of isolates to genus level, cell morphology (Gram reaction, cell shape and arrangement) of all 142 selected colonies were examined under a phase contrast microscope (Olympus CX21FS1, Tokyo, Japan) after Gram staining. Simple biochemical tests (e.g., catalase production, motility, sugar fermentation, and acid and gas production in MRS broth) were also carried out on each presumed LAB isolate according to the method of Dykes (1994). Based on preliminary data obtained, 80 (21 aerobic and 59 anaerobic) of the 142 isolates were selected, preserved in nutrient broth (LAB ‘E’, LAB068, UK) supplemented with 40% glycerol (BDH, Poole, England) and sent to the Agricultural Research Council–Institute for Soil Climate and Water (ARC–ISCW), South Africa for molecular characterization.

#### Molecular Characterization of Isolates

Sixty (21 aerobic and 39 anaerobic) isolates were further selected based on further preliminary tests, for DNA extraction and molecular analysis to determine diversity and phylogeny of the isolates. Genomic DNA extraction was performed according to the protocol described in the ZR Soil Microbe DNA MiniPrep extraction kit (Zymo Research Corporation, Irvine, CA, USA) with modifications. DNA quantification was by the Qubit 2.0 Fluorometer (Q32867, Life Technologies, Grand Island, NY, USA) while verification of DNA quality was performed on 1.5% agarose gel electrophoresis prior to PCR.

All the isolated DNA sequences were used as template to amplify bacterial 16S rRNA genes. PCR amplification was performed with primers 341F (CCTACGGGAGGCAGCAG) and 907R (CCGTCAATTCCTTTAAGTTT). The amplicon sizes were ∼600 bp and were purified and sequenced using the Sanger sequence approach at Inqaba Biotech, South Africa, a commercial service provider. Bidirectional Sanger sequence reads were obtained by standard procedures and the contigs were assembled by the Bio-Edit sequence assembly program (Hall, 1999). Sequence data obtained have been submitted to GenBank under accession numbers: KR812487–KR812544 as well as BWS2E and FWS4B (**Table 1**).

**Table 1:** Bacterial populations identified during steeping of maize for *ogi* production.

Accession numbers	OTUs^1^	Matched species (close relatives from NCBI)	Phylum
KR812487	OTU2	*Lactobacillus paraplantarum* (AJ306297)	Firmicutes
KR812488	OTU4	*Pediococcus acidilactici* (AJ305320)	Firmicutes
KR812489	OTU4	*P. pentosaceus* (AJ305321)	Firmicutes
KR812490	OTU1	*P. acidilactici* (AJ305320)	Firmicutes
KR812491	OTU8	*P. pentosaceus* (AJ305321)	Firmicutes
KR812492	OTU2	*L. paraplantarum* (AJ306297)	Firmicutes
KR812493	OTU8	*P. acidilactici* (AJ305320)	Firmicutes
KR812494	OTU2	*L. paraplantarum* (AJ306297)	Firmicutes
KR812495	OTU8	*P. pentosaceus* (AJ305321)	Firmicutes
KR812496	OTU2	*L. paraplantarum* (AJ306297)	Firmicutes
KR812497	OTU8	*P. pentosaceus* (AJ305321)	Firmicutes
KR812498	OTU8	*P. pentosaceus* (AJ305321)	Firmicutes
KR812499	OTU7	*P. acidilactici* (AJ305320)	Firmicutes
KR812500	OTU3	*Pseudomonas hibiscicola* (AB021405)	Proteobacteria
KR812501	OTU4	*P. acidilactici* (AJ305320)	Firmicutes
KR812502	OTU8	*P. pentosaceus* (AJ305321)	Firmicutes
KR812503	OTU4	*P. acidilactici* (AJ305320)	Firmicutes
KR812504	OTU7	*P. acidilactici* (AJ305320)	Firmicutes
KR812505	OTU5	*Ps. hibiscicola* (AB021405)	Proteobacteria
KR812506	OTU8	*P. acidilactici* (AJ305320)	Firmicutes
KR812507	OTU6	*L. paraplantarum* (AJ306297)	Firmicutes
KR812508	OTU7	*P. acidilactici* (AJ305320)	Firmicutes
KR812509	OTU8	*P. pentosaceus* (AJ305321)	Firmicutes
KR812510	OTU8	*P. pentosaceus* (AJ305321)	Firmicutes
KR812511	OTU9	*Ps. hibiscicola* (AB021405)	Proteobacteria
KR812512	OTU10	*Bacillus thuringiensis* (D16281)	Firmicutes
KR812513	OTU16	*B. mycoides* (AB021192)	Firmicutes
FWS4B	OTU11	*Enterobacter* sp. (KF599041)	Proteobacteria
KR812514	OTU12	*P. acidilactici* (AJ305320)	Firmicutes
BWS2E	OTU13	*Moorella glycerini* (U82327)	Firmicutes
KR812515	OTU14	*B. mycoides* (AB021192)	Firmicutes
KR812516	OTU15	*P. pentosaceus* (AJ305321)	Firmicutes
KR812517	OTU16	*B. mycoides* (AB021192)	Firmicutes
KR812518	OTU16	*B. mycoides* (AB021192)	Firmicutes
KR812519	OTU16	*B. mycoides* (AB021192)	Firmicutes
KR812520	OTU16	*B. mycoides* (AB021192)	Firmicutes
KR812521	OTU8	*P. acidilactici* (AJ305320)	Firmicutes
KR812522	OTU17	*L. paraplantarum* (AJ306297)	Firmicutes
KR812523	OTU18	*P. acidilactici* (AJ305320)	Firmicutes
KR812524	OTU19	*P. acidilactici* (AJ305320)	Firmicutes
KR812525	OTU20	*P. acidilactici* (AJ305320)	Firmicutes
KR812526	OTU21	*P. acidilactici* (AJ305320)	Firmicutes
KR812527	OTU22	*Ps. hibiscicola* (AB021405)	Proteobacteria
KR812528	OTU23	*Ps. hibiscicola* (AB021405)	Proteobacteria
KR812529	OTU24	*Myroides marinus* (GQ857652)	Bacteroidetes
KR812530	OTU25	*Ps. hibiscicola* (AB021405)	Proteobacteria
KR812531	OTU26	*Alcaligenes faecalis* (AJ242986)	Proteobacteria
KR812532	OTU27	*Enterobacter* sp. (KF588515)	Proteobacteria
KR812533	OTU28	*Bordetella avium* (AF177666)	Proteobacteria
KR812534	OTU29	*P. pentosaceus* (AJ305321)	Firmicutes
KR812535	OTU30	*B. mycoides* (AB021192)	Firmicutes
KR812536	OTU31	*M. marinus* (GQ857652)	Bacteroidetes
KR812537	OTU32	*P. acidilactici* (FJ905315)	Firmicutes
KR812538	OTU33	*P. acidilactici* (KT427449)	Firmicutes
KR812539	OTU34	*Acinetobacter bereziniae* (Z93443)	Proteobacteria
KR812540	OTU35	*P. claussenii* (NR_042232)	Firmicutes
KR812541	OTU36	*Enterococcus faecium* (AJ301830)	Firmicutes
KR812542	OTU37	*B. bronchiseptica* (U04948)	Proteobacteria
KR812543	OTU38	*M. marinus* (GQ857652)	Bacteroidetes
KR812544	OTU39	*Ac. bereziniae* (Z93443)	Proteobacteria

*^1^Operational Taxonomic Units*.

The 16S rRNA gene sequences were first affiliated to bacterial taxa using SeqMatch on the Ribosomal Database Project (RDP) website (http://rdp.cme.msu.edu/index.jsp; Cole et al., 2009). Multiple sequence alignments and clustering into operational taxonomic units (OTUs) of the 60 sequences considered herein were performed with Mothur (Schloss et al., 2009), using a 1% dissimilarity level between OTUs (**Table 1**). The evolutionary history was inferred using the Neighbor-Joining method (Saitou and Nei, 1987).

Matched sequences, one for each OTU, were later obtained from the GenBank using the accession numbers. These sequences alongside the OTU representatives were used to construct a library. All sequences were aligned using the multiple sequence alignment software, MAFFT version 7 (Katoh and Standley, 2013). A region of specific data matrice was built on sequence alignment and used to generate combined sequence alignment of multiple regions. Finally, Mega4 software was used to generate a phylogenetic tree consisting of representative OTUs and their close counterparts (matched sequences; Tamura et al., 2007), using the *Aquifex aeolicus* 16S rRNA gene as an outgroup sequence. The percentages of replicate trees in which the associated taxa clustered together in the bootstrap test (1000 replicates) are shown next to the branches (Felsenstein, 1985).

### Analysis of Maize and *Ogi* Samples for Mycotoxins

Prior to batching the maize grains for steeping and *ogi* production (see section on *Source of maize grains and preparation of ogi samples*), 500 g subsample of the grains was randomly taken, milled and quartered. A quarter (125 g) of the milled sample was taken for mycotoxin analysis and triplicate maize samples were analyzed. About 80 g subsample of each pressed *ogi* (see section on *Source of maize grains and preparation of ogi samples*) was also taken and quartered. The maize and *ogi* samples were immediately shipped on dry ice to IFA-Tulln, Austria for mycotoxin analysis. All samples were kept at –20°C at IFA-Tulln until mycotoxin analysis. Mycotoxin analysis of maize and *ogi* samples was performed by liquid chromatography tandem mass spectrometry (LC–MS/MS).

Five grams of each homogenized representative maize or *ogi* sample was weighed into a 50 ml polypropylene tube (Sarstedt, Germany) and extracted with 15 ml acetonitrile/water/acetic acid (79:20:1, v/v/v) for 90 min on a GFL 3017 rotary shaker (GFL, Burgwedel, Germany). Extracts were diluted in extraction solvent and injected into the LC instrument as described in detail by Malachova et al. (2014). Mycotoxins and other microbial metabolites described by Malachova et al. (2014) were screened using a QTrap 5500 LC-MS/MS System (Applied Biosystems, Foster City, CA, USA) equipped with a TurboV electrospray ionization (ESI) source and a 1290 Series UHPLC System (Agilent Technologies, Waldbronn, Germany). Chromatographic separation was performed at 25°C on a Gemini^®^ C_18_-column, 150 mm × 4.6 mm i.d., 5 μm particle size, equipped with a C_18_ security guard cartridge, 4 mm × 3 mm i.d. (all from Phenomenex, Torrance, CA, USA). Positive analyte identification was confirmed by the acquisition of two MS/MS transitions which yielded 4.0 identification points according to commission decision 2002/657/EC. Furthermore, the LC retention time and the intensity ratio of the two MRM transitions agreed with the related values of an authentic standard within 0.1 min and 30% rel., respectively. Further details relating to spiking, recoveries and additional LC–MS/MS parameters are as reported in our previous papers (Warth et al., 2012; Abia et al., 2013a).

### Estimation of Mycotoxin Reduction in *Ogi* Due to Fermentation

In order to estimate percentage reduction of each mycotoxin due to fermentation influenced by fermenter diversity, the percentage difference between mycotoxin levels in the grain and final product (*ogi*) was calculated, taking into consideration the sum of mycotoxin levels lost due to other processes involved in *ogi* production (e.g., washing of grains, mashing and sieving of slurry, and discarding of pomace). Details of the influence of steeping and processing practices on reduction of mycotoxins and other microbial metabolites during *ogi* production will be described elsewhere (Okeke et al., *manuscript in preparation*).

### Data Analysis

SPSS 15.0 for Windows (SPSS, Inc., Chicago, IL, USA) was used for data analyses of (a) occurrence of fermenter species, and (b) mycotoxin reduction levels. Means were separated by the Duncan’s Multiple Range test and tested for significance by one-way analysis of variance at α = 0.05.

## Results and Discussion

### Bacterial Diversity During Steeping of Maize for *Ogi* Production

A total of 142 bacterial isolates were obtained from the steeping processes of both maize varieties; 73 and 69 isolates from white and yellow varieties, respectively. The isolates obtained on PCA (*n* = 51) represented aerobic or facultatively anaerobic species while those on MRS agar (*n* = 91) were LAB and represented obligate or facultative anaerobic homofermentative cocci or heterofermentative cocci and rods. Preliminary identification tests of all the 142 isolates suggested the LAB isolates were Gram-positive, catalase negative and non-motile (Odunfa and Adeyele, 1985; Teniola and Odunfa, 2002; Teniola et al., 2005) and belonged to *Lactobacillus* and *Pediococcus*, while other bacteria belonged to *Bacillus*, *Enterococcus* and a range of rod-shaped bacteria.

**FIGURE 1:**
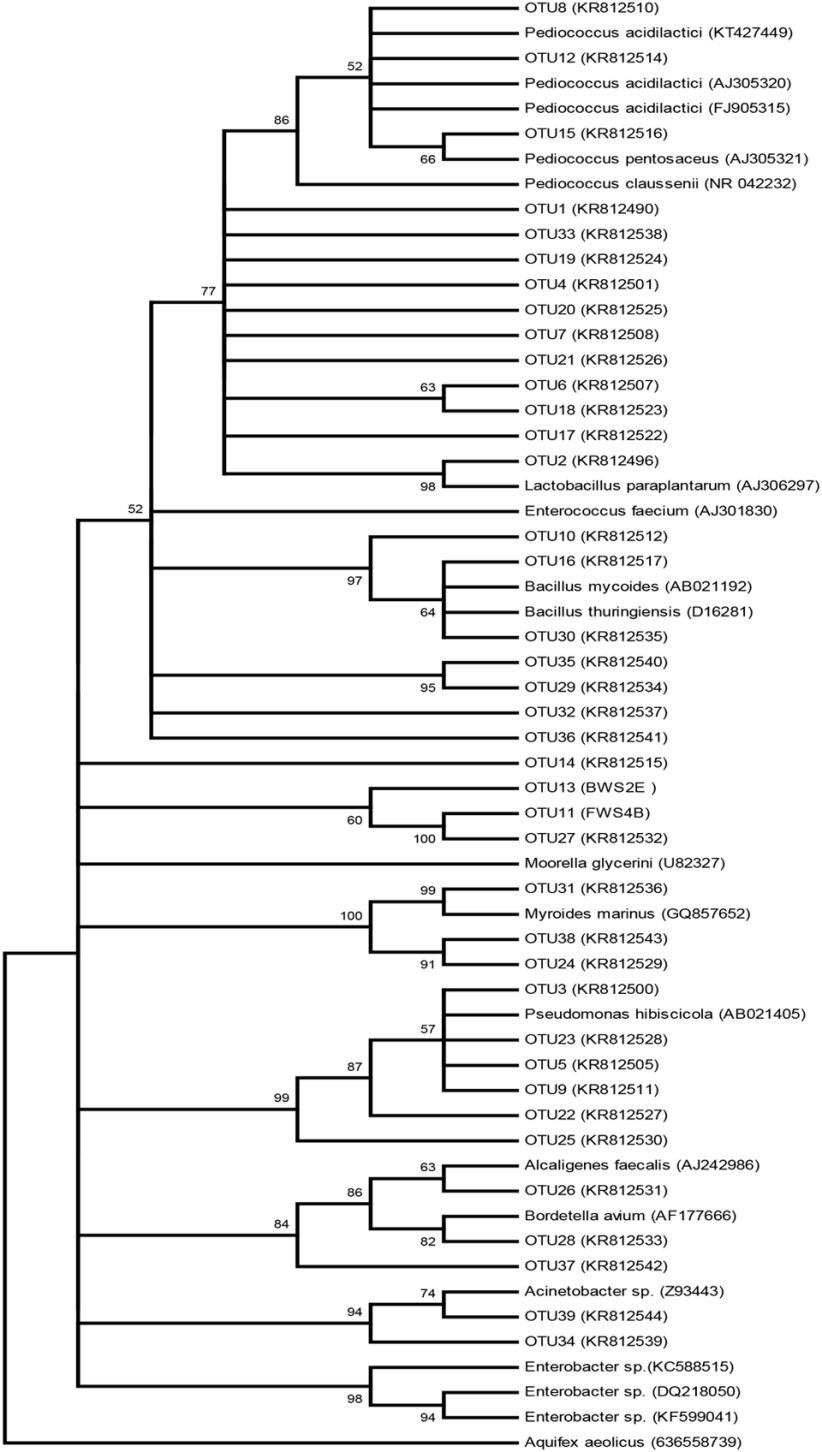
**Phylogenetic tree illustrating major bacterial taxa identified during steeping of maize for *ogi* production and their relatives obtained from the Seqmatch (RDP)**.

**FIGURE 2:**
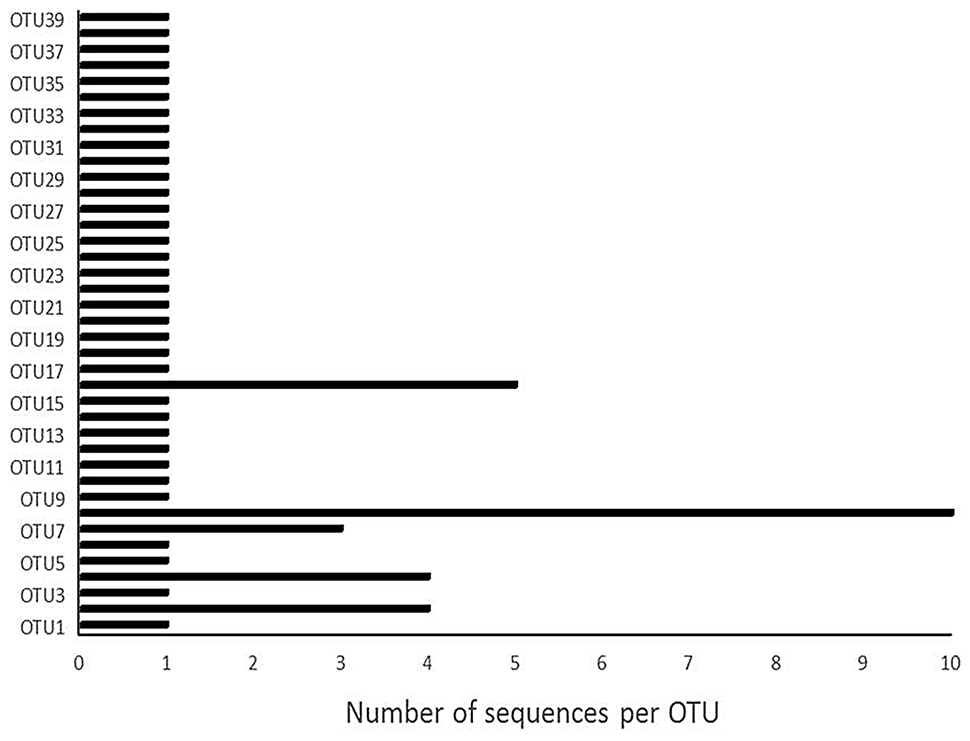
**Operational Taxonomic Units (OTUs) and number of sequences per OTU**.

Our choice of cultivation techniques aided the discrimination of living and dead microorganisms that are able to participate in the fermentation process. However, to ensure proper identification of the bacterial isolates, molecular techniques were employed. Molecular characterization of the isolates clustered them into 39 OTUs representing three phyla, eight families, 10 genera and 15 species (**Table 1**, **Figures 1** and **2**). The families (Alcaligenaceae, Bacillaceae, Enterobacteriaceae, Enterococcaceae, Flavobacteriaceae, Lactobacillaceae, Moraxellaceae, and Xanthomonadaceae) are not shown in the tables or figures.

Among the identified OTUs were four distinct LAB species – *L. paraplantarum*, *P. acidilactici*, *P. claussenii*, and *P. pentosaceus* (**Table 1**, **Figure 2**). Similar spectra and even more species of LAB excluding *L. paraplantarum* and *P. claussenii* have been previously reported in *ogi* made from a range of cereals including guinea-corn, maize, millet, and sorghum (Teniola and Odunfa, 2002; Teniola et al., 2005; Adebayo and Aderiye, 2007; Adebayo-tayo and Onilude, 2008; Oguntoyinbo et al., 2011; Omemu, 2011; Banwo et al., 2012; Oguntoyinbo and Narbad, 2012). *L. plantarum* and various species of *Lactococcus* and *Leuconostoc* were previously reported in maize steep liquor at 24–72 h by Oyedeji et al. (2013). However, this first report of *L. paraplantarum* and absence of *L. plantarum*, *Lactococcus*, and *Leuconostoc* in our study may have been mainly due to selective/biased isolation/subculturing influenced by culture-dependent methods, especially when *L. plantarum* was reported to be predominant during *ogi* production by previous authors who employed molecular tools for species identification (Oguntoyinbo et al., 2011; Banwo et al., 2012; Oguntoyinbo and Narbad, 2012). *L. paraplantarum* was previously found in beer (Curk et al., 1996) and subsequently in *koko* sour water from millet in Ghana (Lei and Jakobsen, 2004).

Among the *Pediococcus* species we found, *P. pentosaceus* had previously been identified in *kenkey* and white maize grains steeped for 24 h for *ogi* (Halm et al., 1993; Teniola et al., 2005) while *P. acidilactici* was also found in *kenkey* and during maize fermentation for *masa* (Oyeyiola, 1990; Halm et al., 1993; Fowoyo and Ogunbanwo, 2010; Sanni and Adesulu, 2013) and recently, in sorghum *ogi* (Banwo et al., 2012). Our study is therefore the first report on association of *P. acidilactici* and *P. claussenii* with *ogi* production from maize. Additional isolates obtained in the present study belonged to *Acinetobacter*, *Alcaligenes*, *Bacillus*, *Bordetella*, *Enterobacter*, *Enterococcus*, *Moorella*, *Myroides*, and *Pseudomonas* (**Table 1**). These genera are known soil bacteria, human, and animal pathogens, and are of no value to fermentation except for *Bacillus, Enterobacter, and Enterococcus* which had been detected during food fermentations in Africa and Asia (Abriouel et al., 2006; Chiang et al., 2006; Oguntoyinbo et al., 2010; Oguntoyinbo and Narbad, 2012).

**FIGURE 3:**
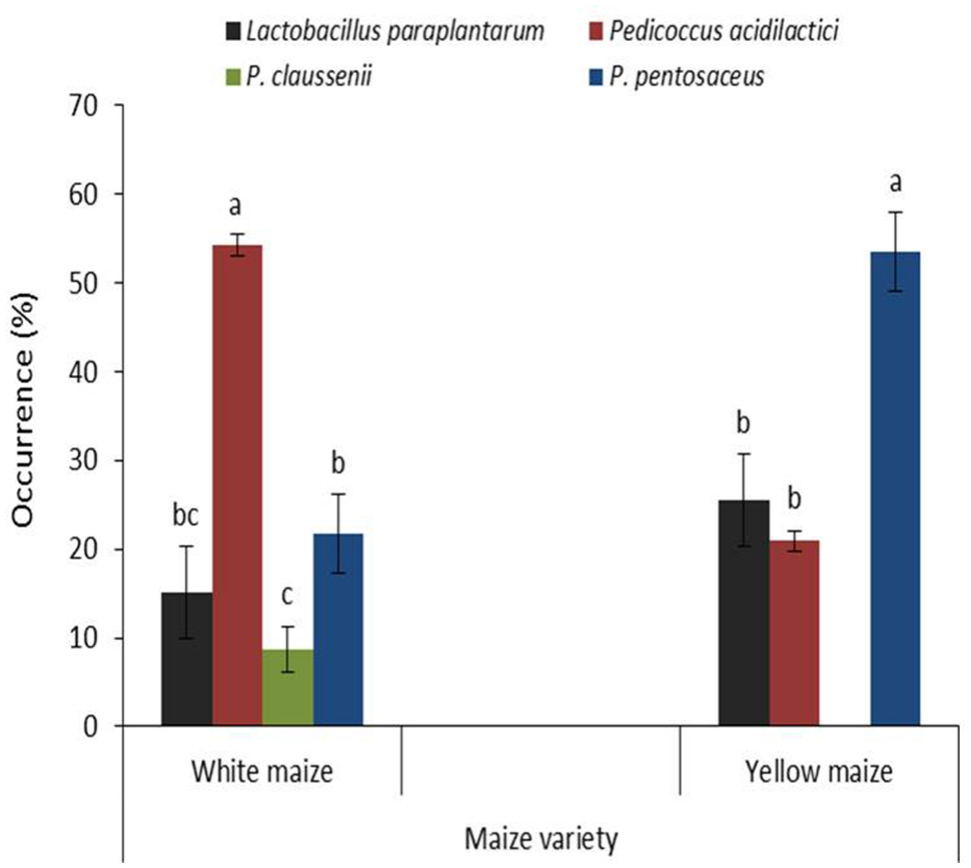
**Occurrence of lactic acid bacteria in steep liquor of two varieties of maize grains fermented for 94 h**. Vertical lines on bars indicate the standard error of mean (α = 0.05). Bars with different alphabets are significantly different by DMRT at α = 0.05.

Maize varietal specific distribution of the LAB species is shown in **Figure 3**. Overall, LAB constituted 62.3–63.0% of all isolated bacteria from steep water of both maize varieties; *Pediococcus* dominated (84.8% in white maize and 74.4% in yellow maize) while the occurrence of *Lactobacillus* was 15.2 and 25.6% in white and yellow maize, respectively. The frequencies of the LAB isolates by OTU cluster in each maize variety are given here: white maize (*Pediococcus* = 20.5%, *Lactobacillus* = 5.1%); yellow maize: (*Pediococcus* = 15.4%, *Lactobacillus* = 2.6%). In terms of species distribution, *Pediococcus acidilactici* (54.3%) and *P. pentosaceus* (53.5%) significantly (*p* < 0.05) predominated over other species in steep water of white and yellow maize varieties, respectively, while *P. claussenii* (8.7%) was found only in white maize (**Figure 3**). Our findings contrast with previous reports on several species of *Lactobacillus* (e.g., *L. delbrueckii* subsp. *bulgaricus*, *L. fermentum*, and *L. plantarum*) predominating in *ogi*, it’s fermentation processes, or in other traditional foods such as fermenting cassava for *garri*, raw milk for *nono*, *massa*, and *wara* (Johansson et al., 1995; Teniola and Odunfa, 2002; Adebayo and Aderiye, 2007; Adebayo-tayo and Onilude, 2008; Fowoyo and Ogunbanwo, 2010; Oguntoyinbo et al., 2011; Omemu, 2011; Banwo et al., 2012; Oguntoyinbo and Narbad, 2012; Obinna-Echem et al., 2013; Oyedeji et al., 2013). This emphasizes the need for more elaborate efforts toward studying the diversity of beneficial natural flora of maize and exploitation of such bacteria for traditional food processes.

### Bacterial Succession During Steeping of Maize for *Ogi* Production

**FIGURE 4:**
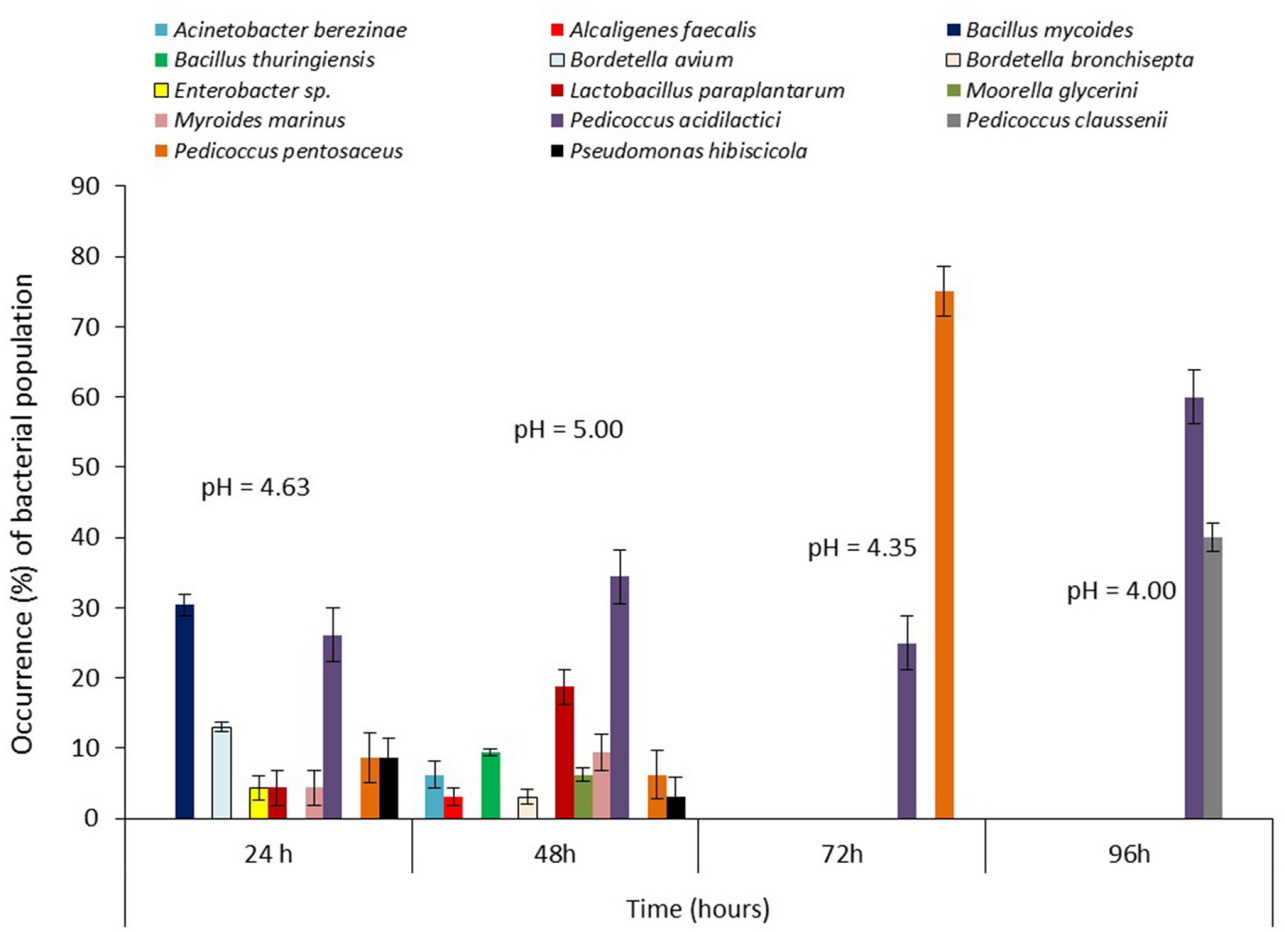
**Changes in bacterial community structure and pH during steeping of white maize grains for *ogi* production**.

**FIGURE 5:**
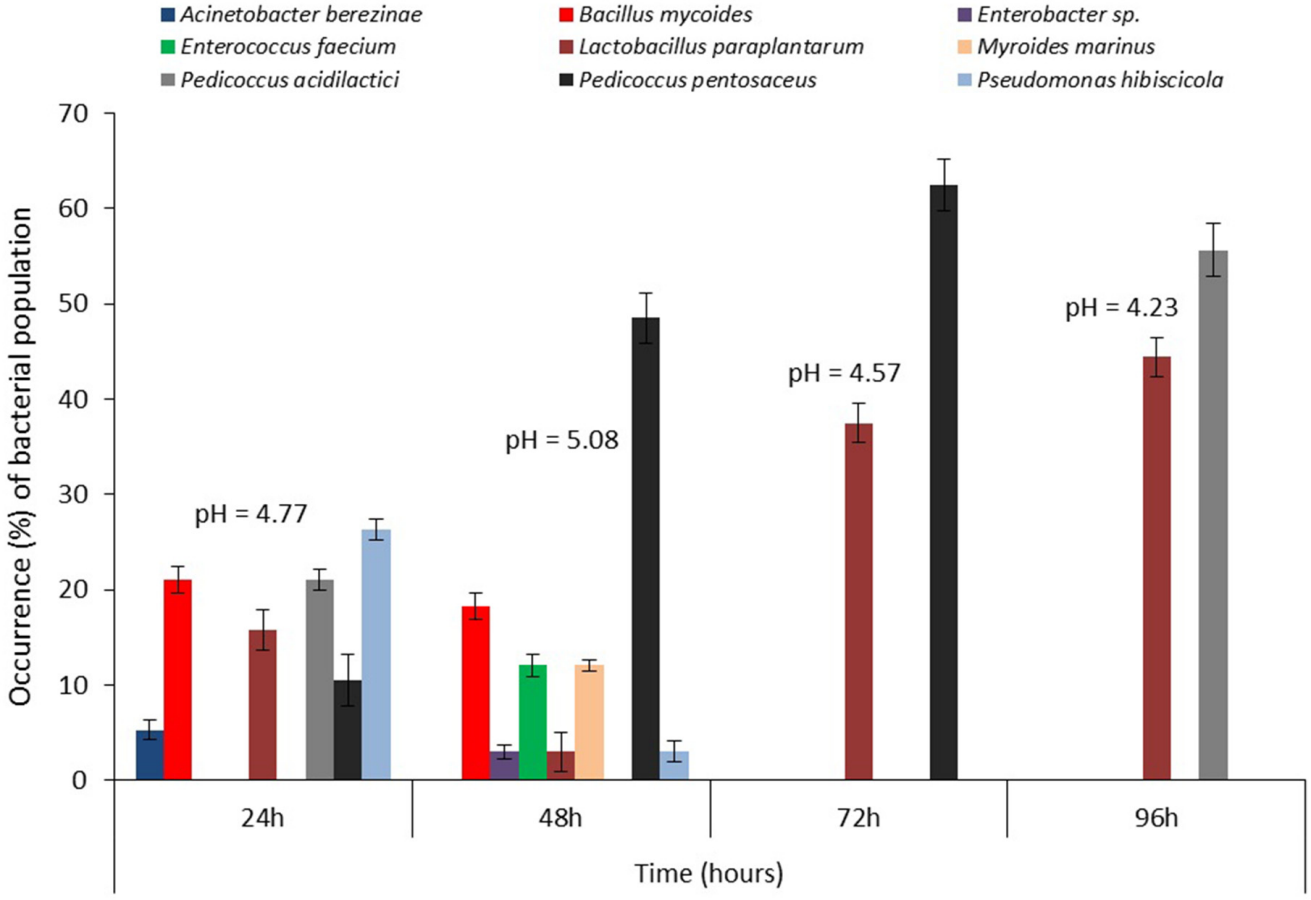
**Changes in bacterial community structure and pH during steeping of yellow maize grains for *ogi* production**.

Molecular analysis strongly indicated succession among the bacterial communities during the steeping/fermentation process of both maize varieties (**Figures 4** and **5**). Succession patterns differed from previous reports (Teniola et al., 2005; Adebayo and Aderiye, 2007; Omemu, 2011; Oyedeji et al., 2013) and also in the two maize varieties though the LAB isolates dominated through the successional periods. For the white maize, *P. acidilactici* occurred at all stages of steeping [pioneered at 24 h (occurrence = 26.1%) and climaxed at 96 h (occurrence = 60%)] while *L. paraplantarum* (4.4–18.8%) and *P. pentosaceus* (6.3–75%) were detected only at seral stages (24–48 h and 24–72 h, respectively; **Figure 4**). *Pediococcus claussenii* (40%) was detected only at the climax stage. In addition, the non-LAB species (3.1–30.4%) were prominent only at the early stages (24–48 h) with *Bacillus mycoides* (30.4%) dominating at the pioneer stage. In the yellow maize, *L. paraplantarum* was detected from pioneer (occurrence = 15.8%) to climax (occurrence = 44.4%) of steeping duration. *Pediococcus pentosaceus* (10.5–62.5%) co-pioneered with *L. paraplantarum*, *P. acidilactici* (21.1%), and the non-LAB species (5.3–26.3%), extending to 72 h before disappearing from the community while *P. acidilactici* was subsequently detected at climax where it dominated (occurrence = 55.6%). Clearly, a wide range of beneficial, opportunistic and potentially pathogenic microbial communities were present and actively interacting in the microenvironment.

The presence and dominance of LAB species such as *L. paraplantarum*, *P. acidilactici*, and *P. pentosaceus* at various steeping times suggest that these and other previously reported LAB species not found in our study are the primary and influential bacterial species involved in the fermentation of maize to *ogi*. These LAB species may therefore be exploited as probiotics and potential starter cultures. The fluctuations of the pH from 4.63 through 5.00 to 4.00 in white maize and from 4.77 through 5.08 to 4.23 in yellow maize steep liquors indicate fluctuations in acid production and release into the microenvironment (Adebayo and Aderiye, 2007; Oyedeji et al., 2013). Furthermore, the extremely low pH (4.00) environment at 96 h of white maize fermentation created by *P. acidilactici* may have led to the entry and stabilization of *P. claussenii* in the succession as this LAB species has only been implicated in spoilage of fermented beverage (beer) due to diacetyl production (Bergsveinson et al., 2012). It was obvious that the non-LAB isolates were excluded from the environment marked by higher acidity (after 48 h) due to intense competition from LAB isolates capable of producing antibacterial compounds such as hydrogen peroxide, diacetyl, and bacteriocins during the process of sugar utilization to release acids (Teniola and Odunfa, 2002; Ogunbanwo et al., 2003, 2004; Adebayo and Aderiye, 2007; Dike and Sanni, 2010; Oyedeji et al., 2013), though these chemical products were not studied here.

### Occurrence of Major Mycotoxins in White and Yellow Maize Grain

**FIGURE 6:**
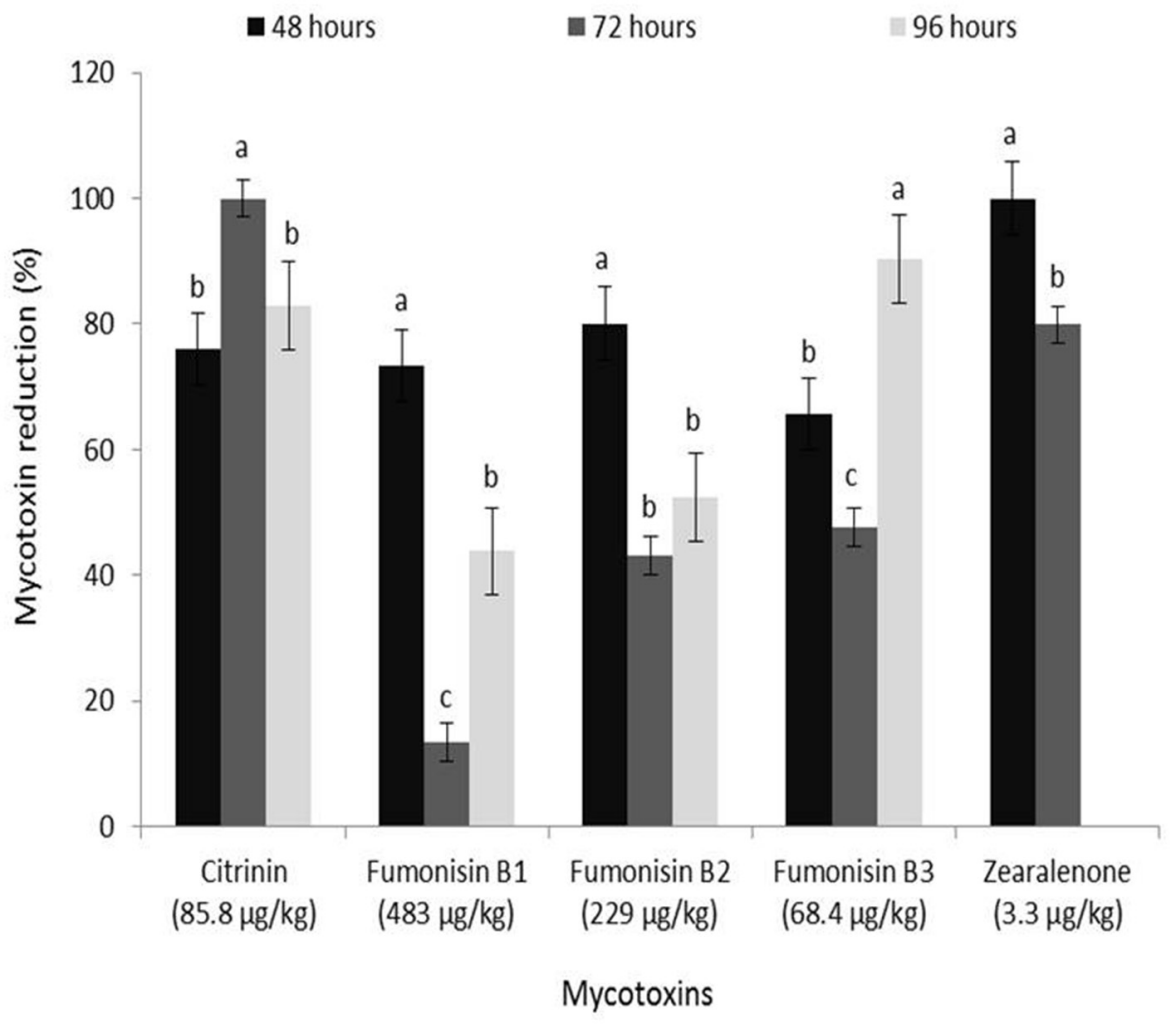
**Reduction (%) of mycotoxins in freshly fermented *ogi* due to fermentation during steeping of white maize grains**. Concentrations given on x-axis indicate mycotoxin levels in raw maize grains before steeping. Cyclopiazonic acid (23.5 μg/kg) was reduced by 100% at all time intervals. Vertical lines on bars indicate the standard error of mean (α = 0.05). Bars with different alphabets are significantly different by DMRT at α = 0.05.

**FIGURE 7:**
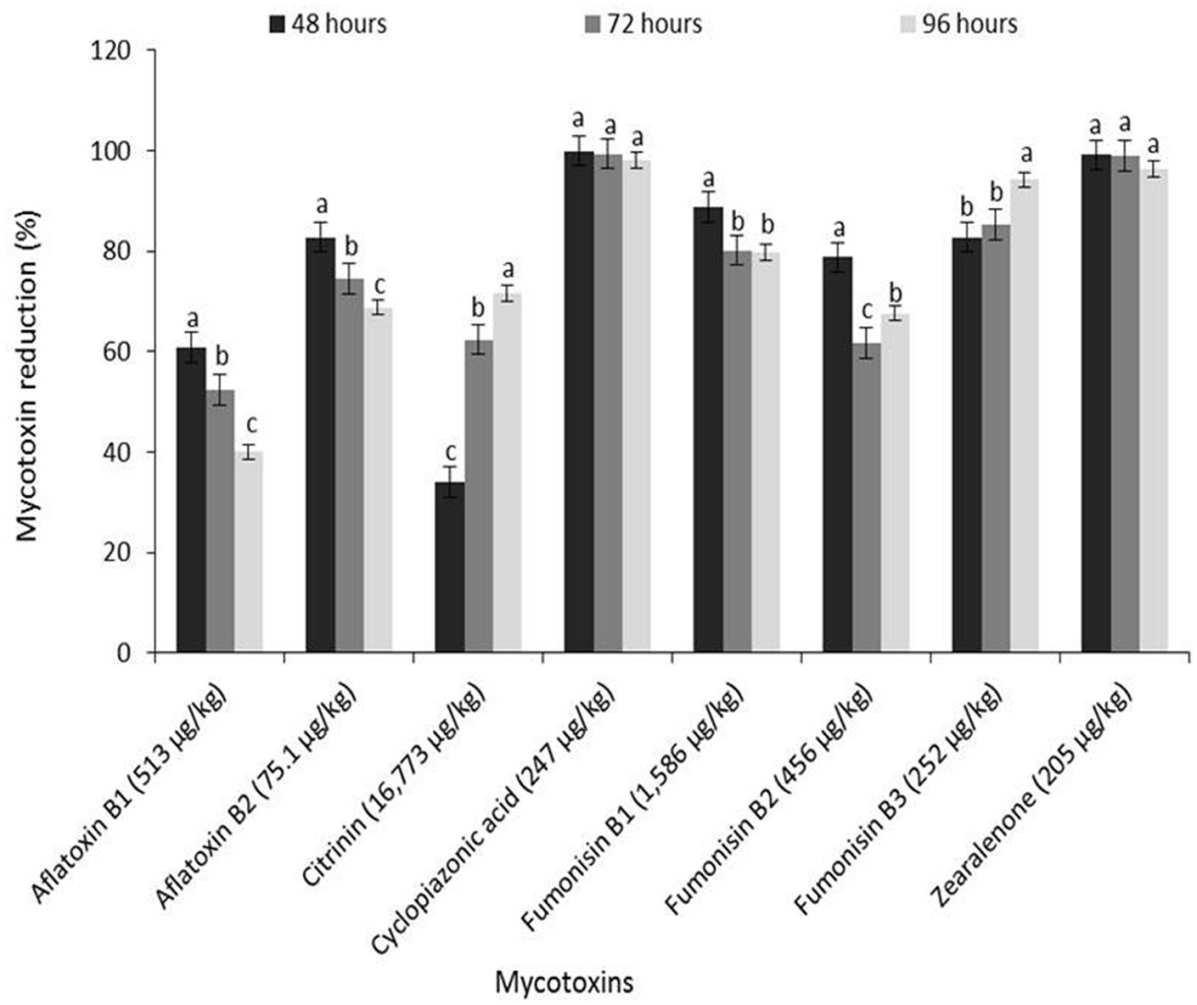
**Reduction (%) of mycotoxins in freshly fermented *ogi* due to fermentation during steeping of yellow maize grains**. Concentrations given on x-axis indicate mycotoxin levels in raw maize grains before steeping. Aflatoxin M_1_ (22.7 μg/kg) was reduced by 100% at all time intervals. Vertical lines on bars indicate the standard error of mean (α = 0.05). Bars with different alphabets are significantly different by DMRT at α = 0.05.

The concentrations of mycotoxins quantified in unsteeped grains of the two maize varieties are shown in **Figures 6** and **7**. Seven mycotoxins [aflatoxin B_1_ (AFB_1_) = 0.60 μg/kg; citrinin (CIT) = 85.8 μg/kg; cyclopiazonic acid (CPA) = 23.5 μg/kg; fumonisin B_1_ (FB_1_) = 483 μg/kg; fumonisin B_2_ (FB_2_) = 229 μg/kg; fumonisin B_3_ (FB_3_) = 68.4 μg/kg; zearalenone (ZEN) = 3.3 μg/kg] were found in the white grain (**Figure 6**), while nine mycotoxins [AFB_1_ = 513 μg/kg; aflatoxin B_2_ (AFB_2_) = 75.1 μg/kg; aflatoxin M_1_ (AFM_1_) = 22.7 μg/kg; CIT = 16,800 μg/kg; CPA = 247 μg/kg; FB_1_ = 1,586 μg/kg; FB_2_ = 456 μg/kg; FB_3_ = 252 μg/kg; ZEN = 205 μg/kg] occurred in the yellow variety (**Figure 7**). Mycotoxin levels in the yellow grains stored for about six months were at least twofold higher than the levels in the white variety barely stored for a month. Citrinin, a mycotoxin produced by *Aspergillus* and *Penicillium*, is reported here for the first time in Nigerian maize and *ogi* (see section on *Reduction of mycotoxins in ogi influenced by fermentation*) though it has previously been reported in maize from India and fermented maize from Ghana (Vella et al., 1995; Janardhana et al., 1999). The spectrum and levels of the other mycotoxins reported in this study, especially the increased levels in stored yellow maize grain, are similar to those previously reported across sub-Saharan Africa. This provides further evidence that mycotoxin contamination of maize and especially their accumulation under poor storage conditions remain a major food safety challenge warranting urgent attention in many countries in SSA (Udoh et al., 2000; Kankolongo et al., 2009; Ncube et al., 2011; Warth et al., 2012; Abia et al., 2013a; Adetunji et al., 2014a).

### Reduction of Mycotoxins in *Ogi* Influenced by Fermentation

Estimated percentage reductions of mycotoxins in white and yellow *ogi* due to fermentation are shown in **Figures 6** and **7**. Estimates were based on percentage differences between mycotoxin levels in the grain and *ogi*, taking into consideration the sum of mycotoxin levels lost due to other processes involved in *ogi* production. Details on the influence of steeping and processing practices on reduction of mycotoxins and other microbial metabolites during *ogi* production will be described elsewhere (Okeke et al., *manuscript in preparation*). The level of AFB_1_ (0.60 μg/kg) in white maize was very low to determine reduction, hence this was excluded from the percentage reduction estimations. On the other hand, CPA and AFM_1_ were reduced to levels below the limits of detection (LOD; CPA: <25 μg/kg; AFM_1_: <0.4 μg/kg, equivalent to 100% reduction) by fermentation of white and yellow maize, respectively, into *ogi* at all time intervals. Additionally, CIT and ZEN in white maize were completely lost (levels detected in *ogi* were <LOD; CIT ≤ 2.5 μg/kg, ZEN ≤ 0.05 μg/kg) during steeping for 72 and 48 h, respectively, while levels of CPA in yellow maize completely diminished (<LOD) at 48 h of steeping (**Figures 6** and **7**). This is the first report of AFM_1_, CIT and CPA degradation/loss due to fermentation in any traditional cereal-based fermented food product.

There were significant (*p* < 0.05) differences in the percentage reductions of mycotoxins across steeping time intervals (48–96 h) for both maize varieties though no time-dependent reduction of the mycotoxins was recorded for the white maize (**Figure 6**). For the yellow maize, percentage reduction of CIT and FB_3_ significantly (*p* < 0.05) increased as steeping time increased while for other toxins (AFB_1_, AFB_2_, FB_1_, and FB_2_) percentage reduction dropped significantly (*p* < 0.05) as steeping time increased from 48 to 96 h (**Figure 7**). Reduction levels of CPA (100–98.1%) and ZEN (99.2–96.4%) dropped insignificantly from 48 to 96 h. Generally, steeping of maize for 48 h drastically reduced fumonisin (FB_1_, FB_2_, and FB_3_) levels in both varieties (white maize: 65.7–80.1%; yellow maize: 78.7–88.8%) as well as aflatoxins (AFB_1_: 60.8%; AFB_2_: 82.8%) and ZEN (99.2%) in the yellow variety.

The extremely high levels of AFB_1_, CIT, CPA, FB_1_, and ZEN reported in raw maize samples in this study raise questions about the safety of consuming maize-based foods contaminated by mycotoxins. These mycotoxins exert diverse individual or synergistic toxicological effects (hepatotoxicity, nephrotoxicity, genotoxicity, teratogenicity, and immunotoxicity) on human and animal systems (Vella et al., 1995; Janardhana et al., 1999; Bondy and Pestka, 2000; Council for Agricultural Science Technology [Cast], 2003; Flajs and Peraica, 2009). However, the remarkable reduction of all the mycotoxins especially after 48 h of steeping proves that fermentation mediated by natural maize flora reduces mycotoxin levels of *ogi*, thus making it a safer food for consumption than its parent maize material. For fermentation to be very effective in producing foods with the safest levels of mycotoxins it is important to ensure that mycotoxin control begins from the field through post-harvest (storage and handling); this will lead to having unsteeped maize with low mycotoxin levels.

Some mycotoxins (e.g., AFB_1_, FB_1_, and ZEN) have been shown to be degraded to various extents by fermentation bacteria or bio-transformed during fermentation processes (Mokoena et al., 2005; Shetty and Jespersen, 2006; Oluwafemi and Da-Silva, 2009; Cho et al., 2010; Nyamete, 2013; Ezekiel et al., 2015; Zhao et al., 2015) and we observed same. However, our study contradicts the reports of Fandohan et al. (2005) and Mokoena et al. (2006) who reported very low reduction levels of 18–28% for AFB_1_ under spontaneous fermentation conditions and suggested increased but insignificant toxin reduction with prolonged fermentation time. The higher reduction (>80%) observed for ZEN at all time intervals in contrast to the lower levels (∼45%) previously reported by Zhao et al. (2015) when strains of *L. plantarum* were used to remove ZEN from MRS medium, may indicate that mycotoxin degradation during fermentation may either be strain specific or require synergistic interaction of more than one species/strain. The fact that non-LAB species (e.g., *Bacillus subtilis*) has been implicated in ZEN degradation (up to 99% of 1 mg/kg after 24 h) in liquid medium (Cho et al., 2010) supports our findings of higher reduction at 48 h in both maize varieties. At 48 h, bacterial population was highest in both maize varieties (occurrence: white maize = 32/73, yellow maize = 33/69) and species diversity in the microenvironment included LAB and non-LAB species including numerous colonies of *Bacillus* species. In our study, we did not find any bio-transformation product of ZEN (e.g., β-zearalenol – a less estrogenic form) in contrast to reports from Mizutani et al. (2011) and Ezekiel et al. (2015) which showed that fermentation, especially submerged, leads to β-zearalenol formation. An assumption may be that the product released was further degraded by specialized bacteria – a finding that requires further investigation.

Citrinin and FB_3_ reduction levels increased with prolonged fermentation time while levels of other mycotoxins (including AFB_1_) reduced. This may be linked to specialization by bacteria – it is most likely that the LAB isolates which dominated the latter stages of the successional chain were fully responsible for CIT and FB_3_ degradation or detoxification; this needs to be proven. However, the binding of mycotoxins (e.g., aflatoxins) to the surface of *Lactobacillus* species (Haskard et al., 2001; Oluwafemi and Da-Silva, 2009) or reformation of aflatoxins, for example, in increased acidic conditions as explained by Kpodo et al. (1996) may have been the underlying factor of the lower reduction of some mycotoxins as fermentation days extended. Cho et al. (2010) and Zhao et al. (2015) suggested that the viable cell count of bacteria influences toxin detoxification, while Guan et al. (2005) reported the involvement of bacterial peptidoglycans in binding foreign matter in order to confer protection against infections. In view of these suggestions and the fact that Cho et al. (2010) and other authors reported the involvement of non-LAB species and some fungi in mycotoxin detoxification, we propose that mycotoxins (excluding aflatoxins) whose reduction levels decreased with prolonged fermentation were most likely due to more diverse bacteria with mycotoxin-binding capacity existing in the early stages of succession. As one moves up the successional chain toward climax, the diversity of bacteria in the microenvironment reduces which influences effective binding of mycotoxins; hence decreased reduction levels. Our reports on reduction levels of aflatoxins being decreased with increased fermentation time fully agrees with the idea on acidification of the environment which interferes with aflatoxin reduction since pH of the steep liquor dropped drastically (Kpodo et al., 1996).

## Conclusion

The combined application of culture-dependent and molecular tools in this study has provided verified information on the community structure of bacteria playing successive roles during steeping of maize for *ogi* production. This has also translated into tracing the relationship between bacterial diversity and mycotoxin reduction under natural fermentation of maize to *ogi*. Mycotoxin levels in the unsteeped maize grains which were quite high were drastically and significantly reduced during the steeping/fermentation process mediated by diverse bacterial communities including fermenters. In view of our findings, *ogi* may be a relatively safe food for human consumption in terms of mycotoxin contamination. The present study identified knowledge gaps worth investigating and, therefore, propose the following: (a) a comprehensive study of the diversity and successional pattern of bacteria/microbes from steeping to souring, and how these influence mycotoxin transfer from raw maize to soured *ogi*; (b) study to understand which specific bacterial/microbial populations degrade or detoxify specific mycotoxins under natural fermentation conditions using labeled mycotoxins and metabolomics tools. This will enhance selection of appropriate starter cultures capable of optimal fermentation while at the same time, detoxifying mycotoxins.

## Author Contributions

CNE conceived and designed the study. COE, MS, CCN, and SKD contributed to the design of the study. CAO, CCN, and MS carried out the experiment/lab work in Nigeria, South Africa and Austria. CNE, COE, SKD, RAA, and RK supervised the experiment/lab work. CNE, CCN, MS, and RAA analyzed the data. CAO, CNE, and CCN drafted the manuscript. All authors critically revised, fine-tuned, and approved the final draft manuscript.

## Conflict of Interest Statement

The authors declare that the research was conducted in the absence of any commercial or financial relationships that could be construed as a potential conflict of interest.
